# Supplementary material for: The way back home: The invisible burden of the emergency healthcare services
Source: PLoS One. 2024 May 8;19(5):e0298933. doi: 10.1371/journal.pone.0298933 (PMC11078431; doi:10.1371/journal.pone.0298933)
Supplement: Attachment — Submitted filename: revision letter (31JAN) PONE-D-23-26422.docx [file pone.0298933.s003.docx]

MD. Mustafa Enes DEMİREL

Department of Emergency Medicine,

Faculty of Medicine

Bolu Abant Izzet Baysal University University, Bolu, TURKEY

**31 Jan 2023**

Dear Dr. Burak Katipoğlu

Academic Editor

PLOS ONE

We have completed the major revision of our MS entitled “The way back to home: The invisible burden of the emergency healthcare services”. We responded to Reviewers’ comments, and yellow highlighted these in the MS. We believe the MS has improved now.

However, should you require any further information, please do not hesitate to contact me at the above address.

Yours sincerely,

Mustafa Enes Demirel, MD.

Comments and Answers

Reviewer #4: Dear author

Thank for your inteesting to journal. My suggestions are below

1. Although you said you would give the results with the mean and standard deviation in the material section, you gave the results with the median ( percent 25-75%) in the results section. Please correct the relevant sentence in the method section.

-We have added the necessary explanation;

For variables that did not exhibit a normal distribution, the interquartile range (IQR) was used.

Reviewer #5:

1. The cost analysis stated in the study should be stated in dollars, which is the international currency.

Dear reviewer,

The exchange rate of the dollar in our country is not stable. However, healthcare service costs are fixed in Turkish lira. Due to the fluctuating USD-TL exchange rate, we believe that expressing the costs in USD for this study might lead to misinterpretation by the reader. Nevertheless, we aimed to provide readers with an insight in terms of dollars by presenting relevant information on the dollar exchange rates for the respective years below the table.

2. In the discussion section of the study, statistical data were used as numerical values. These data have already been mentioned in the results section. Numerical data from other articles should not be used. Instead, attention should be paid to the academic expression language expressing numerical values and corrected accordingly.

In the discussion section of the study, numerical statistical data were removed. The numerical values representing data from other articles were revised with attention to academic language.
